# Induction of NEDD8-conjugating enzyme E2 UBE2F by platinum protects lung cancer cells from apoptosis and confers to platinum-insensitivity

**DOI:** 10.1038/s41419-020-03184-4

**Published:** 2020-11-12

**Authors:** Lisha Zhou, Jin Zhu, Wangyang Chen, Yanyu Jiang, Tao Hu, Yinxia Wang, Xiaoling Ye, Mengxi Zhan, Chenghao Ji, Zhuoming Xu, Xinran Wang, Yuanlong Gu, Lijun Jia

**Affiliations:** 1 Department of Basic Medical Science, Medical College, Taizhou University, 318000 Taizhou, Zhejiang China; 2 Department of Surgical Oncology, Jiangxi Cancer Hospital, 330029 Nanchang, Jiangxi China; 3 Cancer Institute of Traditional Chinese Medicine, Longhua Hospital, Shanghai University of Traditional Chinese Medicine, 200032 Shanghai, China; 4 Taizhou Municipal Hospital, Clinical Medical College, Taizhou University, 318000 Taizhou, Zhejiang China

**Keywords:** Chemotherapy, Apoptosis

## Abstract

Platinum is a widely used first-line chemotherapy in treating non-small cell lung cancer of adenocarcinoma. Unfortunately, platinum resistance leads to relapse and therapeutic failure, enabling the development of platinum-sensitization strategies to be of great clinical significance. Here, we report that the upregulation of the NEDD8-conjugating enzyme UBE2F is an important way for lung cancer cells to escape platinum-induced cell apoptosis, which confers to insensitivity to platinum-based chemotherapy. Mechanistically, platinum treatment impairs the complex formation for proteasome-mediated UBE2F degradation, evidenced by the weaker association between UBE2F and Ring-box protein 1 (RBX1), an essential component of Cullin-Ring E3 ligases (CRLs), thus leading to the accumulation of UBE2F. The accumulated UBE2F promotes the neddylation levels and activity of Cullin5, in accord with the lower expression of pro-apoptotic protein NOXA, a well-known substrate of Cullin-Ring E3 ligase 5 (CRL5). Additionally, knockout of UBE2F significantly sensitizes lung cancer cells to platinum treatment by enhancing the protein levels of NOXA and subsequently promoting cell apoptosis. Our observations uncover a previously unknown regulatory mechanism of UBE2F stability upon platinum chemotherapy and suggest that UBE2F might be a novel therapy target for sensitizing lung cancer cells to platinum-based chemotherapy.

## Introduction

Lung cancer is the most common human malignancies and remains the leading cause of cancer-related deaths worldwide^1,2^. At present, non-small cell lung cancer (NSCLC) of adenocarcinoma is the most common histological type of lung cancer^3^. Despite improved treatments, NSCLC still has a poor prognosis and the overall 5-year survival rate after diagnosis remains at a very low level^4^. Surgery is an appropriate treatment option for patients with early NSCLC, but platinum-based chemotherapy remains the cornerstone of the treatment in advanced cases without treatable oncogenic alterations^5^. Unfortunately, the further application of platinum-based chemotherapy is seriously limited by the development of drug resistance^6^. Therefore, a better understanding of the molecular mechanism underlying platinum-based chemotherapy resistance and the development of drugs that enhance chemosensitivity is crucial to improving the survival outcomes and quality of life for NSCLC patients.

Neddylation is a process that conjugates a ubiquitin-like molecule NEDD8 (neuronal precursor cell-expressed developmentally down-regulated protein 8) to a lysine residue of the substrate protein and regulates many stages of tumor development including tumorigenesis and metastasis^7,8^. Neddylation is a three-step enzymatic cascade reaction, catalyzed by NEDD8-activating enzyme E1 (NAE, a heterodimer of NAE1/APPBP1 and NAEβ/UBA3), NEDD8-conjugating enzyme E2 (UBE2M or UBE2F), and substrate-specific NEDD8-E3 ligases^9–11^. The best-characterized physiological substrates of neddylation are cullin family members, including CUL-1, 2, 3, 4A, 4B, 5, 7, and 9^12^. Each cullin protein is a scaffold subunit of Cullin-RING E3 ligase (CRL), which controls the degradation of about 20% of proteasome-regulated proteins, including transcription factors, tumor suppressors, and onco-proteins^12–14^. Intensive studies have proven that NEDD8 and enzymes of the neddylation pathway (e.g., NAE1/UBA3, UBE2M/UBE2F, and NEDD8-E3 ligases) are often overexpressed in multiple human cancers, which are associated with tumor progression and predict poor patient survival^7,15^. Neddylation inhibition suppresses tumor cell growth by inducing apoptosis, senescence, and autophagy, leading to the validation of the neddylation pathway as a promising anticancer strategy^7,15^.

In mammalian cells, there are two independent NEDD8-conjugating enzyme E2s, UBE2M, and UBE2F, with distinct functions^16–18^. UBE2M pairs with RBX1 to regulate neddylation of CUL-1, −2, −3, −4A, and −4B, whereas UBE2F is highly specific to the neddylation of RBX2-associated CUL-5^18^. The transcriptome analysis of UBE2M vs UBE2F knockdown showed that the gene expression patterns are largely non-overlapping^18^. UBE2M knockdown leads to the accumulation of tumor-suppressive CRLs substrates (such as p21 and p27) to induce cell-cycle arrest and inhibit tumor growth and metastasis^19,20^, and significantly sensitizes cell to ionizing radiation (IR)^21^ and DNA-damaging agents^20^. Unlike the extensive research on UBE2M, UBE2F is rarely reported. Recent studies reported that UBE2F couples with RBX2 E3 to induce CUL-5 neddylation and activation, resulting in poly-ubiquitylation of pro-apoptotic protein NOXA for proteasomal degradation and apoptosis inhibition of lung cancer cells, thus acting as an anti-apoptotic protein^22,23^.

Drug resistance of NSCLC to platinum-based chemotherapy is mainly due to the inactivation of apoptotic pathways^24^. Therefore, as the apoptotic regulatory protein, the role of UBE2F in the chemosensitivity of lung cancer cells to platinum is worth further investigation. This study is directed toward understanding how UBE2F responds to platinum-based chemotherapy and then allows for the platinum-insensitivity of lung cancer cells. Our results uncover a novel mechanism of UBE2F stability regulation by CRL complex upon platinum-based chemotherapy and suggest a potential therapeutic strategy of enhancing platinum chemosensitivity through targeting UBE2F.

## Materials and methods

### Cell culture and reagents

293T cells, human lung cancer cell lines A549 and H1299, and human breast cancer cell line MB231 were obtained from the American Type Culture Collection. ES-2 (an ovarian cancer cell line) and SW480 (a colon cancer cell line) were given by University Shanghai Cancer Center. Cells were cultured in Dulbecco’s modified Eagle’s medium (DMEM, hyclone, Logan, UT), containing 10% fetal bovine serum (Biochrom AG, Berlin, Germany) and 1% penicillin–streptomycin solution at 37 °C with 5% carbon dioxide.

For CHX-chase experiments, cells were treated with 50 μg/mL CHX (Sigma, C4859) in combination with 10 μM MG-132 (Sigma, M7447) or DMSO for indicated time points; cells were treated with 50 μg/mL CHX (Sigma, C4859) in combination with 48 h treatment of 4 μΜ ciaplatin (Selleck, S1166), 20 μg/mL carboplatin (Selleck, S1215) or PBS for indicated time points.

### Antibodies and plasmids

Antibodies specific to C-PARP (Cell Signaling Technology, 5625), Cullin1 (Abcam, 75817), Cullin2 (Abcam, 166917), Cullin3 (Cell Signaling Technology, 2759), Cullin4A (Cell Signaling Technology, 2699), Cullin4B (Proteintech, 12916-1-AP), Cullin5 (Abcam, 184177), NAE (Cell Signaling Technology, 14321), NOXA (Cell Signaling Technology, 14766), P27 (Cell Signaling Technology, 3686), RBX1 (Abcam, 133565), UBA3 (Abcam, 124726), UBE2F (Abcam, 12932), and UBE2M (Abcam, 109507) were purchased commercially.

### Generation of stable cell lines by CRISPR/Cas9 system

For packaging lentivirus used in UBE2F knockdown, two guide RNA sequences specifically against UBE2F were inserted into vector lenti-guide-puro, respectively. 293T cells were co-transfected with lentiviral vectors lenti-guide-puro (4 μg) and packaging vectors AGP091 (3.0 μg) and AGP090 (1.2 μg). Forty-eight hours after transfection, the viral supernatants were collected, filtered, and infected A549 cells. Polybrene (Sigma-Aldrich, St. Louis, MO) was added into viral supernatant at the concentration of 10 μg/mL. Six hours after incubation, the viral supernatant was replaced with normal DMEM with 10% FBS.

### Cell survival assay

Human lung cancer cells A549 were seeded in 6-well plates at a density of 50,000 cells/well, followed by treatment of low concentration of cisplatin (0.4 μΜ) or carboplatin (2 μg/mL), and then cell numbers were trypsinized and counted every 1–2 days over a 7-day period by using Nexcelom. For the treatment of high concentration cisplatin or carboplatin, A549 were seeded in 6-well plates at a density of 500,000 cells/well. After incubation with cisplatin (4 μΜ) or carboplatin (20 μg/mL) for 48 h, the remaining living cells were trypsinized and counted by Nexcelom. Representative results of three independent experiments with similar trends are presented.

### Gene silencing using small interfering RNA (siRNA)

Cells were transfected with siRNA oligonucleotides and synthesized by GENEPHARMA (Shanghai, China) using Lipofectamine 2000. The sequences of the siRNA were as follows:

RBX1: 5′-GACTTTCCCTGCTGTTACCTAATT-3′;

RBX2: 5′-GAGGACTGTGTTGTGGTCT-3′;

Cullin1: 5′-CUAGAUACAAGAUUAUACAUGCGG-3′;

Cullin2: 5′-GCACAAUGCCCUUAUUCAA-3′;

Cullin3: 5′-TTGACGTGAACTGACATCCACATTC-3′;

Cullin4A: 5′-GAAGAUUAACACGUGCUGGTT-3′;

Cullin4B: 5′-AAGCCUAAAUUACCAGAAA-3′;

Cullin5: 5′-CUACUGACUCUGAGAAAUA-3′;

NOXA: 5′-GTAATTATTGACACATTTC-3′.

### Immunoprecipitation and western blotting

For Flag immunoprecipitations, cells were lysed in an ice-cold NP-40 buffer (50 mM Tris–HCl (pH 7.4), 150 mM NaCl, 0.1% NP-40, and protease inhibitors). The cell lysate was incubated with anti-Flag M2 affinity resin (Sigma-Aldrich, A2220) for 3 h at 4 °C, washed three times with ice-cold NP-40 buffer, and analyzed by SDS–PAGE and immunoblotting. Western blot analysis was carried out according to standard methods.

### Animal experiments

Animal studies were performed in accordance with animal protocol procedures approved by the Institutional Animal Care and Use Committee of Taizhou University. Nude mice (nu/nu, female, 6–8 weeks old), purchased from the Shanghai Experimental Animal Center (Shanghai, China), were injected subcutaneously with A549 stable cells with endogenous UBE2F knockout (2 × 10^6^ cells). When the size of the tumor reaches about 20 mm^2^, mice were intraperitoneally injected with saline, cisplatin (4 mg/kg, once every 4 days, three cycles), or carboplatin (25 mg/kg, once every 2 days, six cycles). Tumor size was measured by a vemier caliper and calculated as (length × width). Tumor weight was measured by analytical balance. No blinding was performed for the animal experiments.

### Collection of lung tissues and clinicopathological characteristics of patients

Tumorous lung tissues of patients were collected by the Taizhou Municipal Hospital of Taizhou university. A physician obtained informed consent from the patients. The procedures related to human subjects were approved by the Taizhou Municipal Hospital of Taizhou university. Direct immunoblotting for these samples was performed as described above.

### Statistical analyses

All data are presented as the mean ± standard deviation from at least three independent experiments. The statistical significance of differences between groups was assessed using the Graph Pad 5 software (Graph Pad Software, San Diego, CA, USA). The unpaired two-tailed *t*-test was used for the comparison of parameters between groups. For all the tests, three levels of significance (*P < 0.05, **P < 0.01, ***P < 0.001) were used.

## Results

### Platinum treatment causes the accumulation of UBE2F via the inhibition of proteasome-mediated protein degradation

**Fig. 1:**
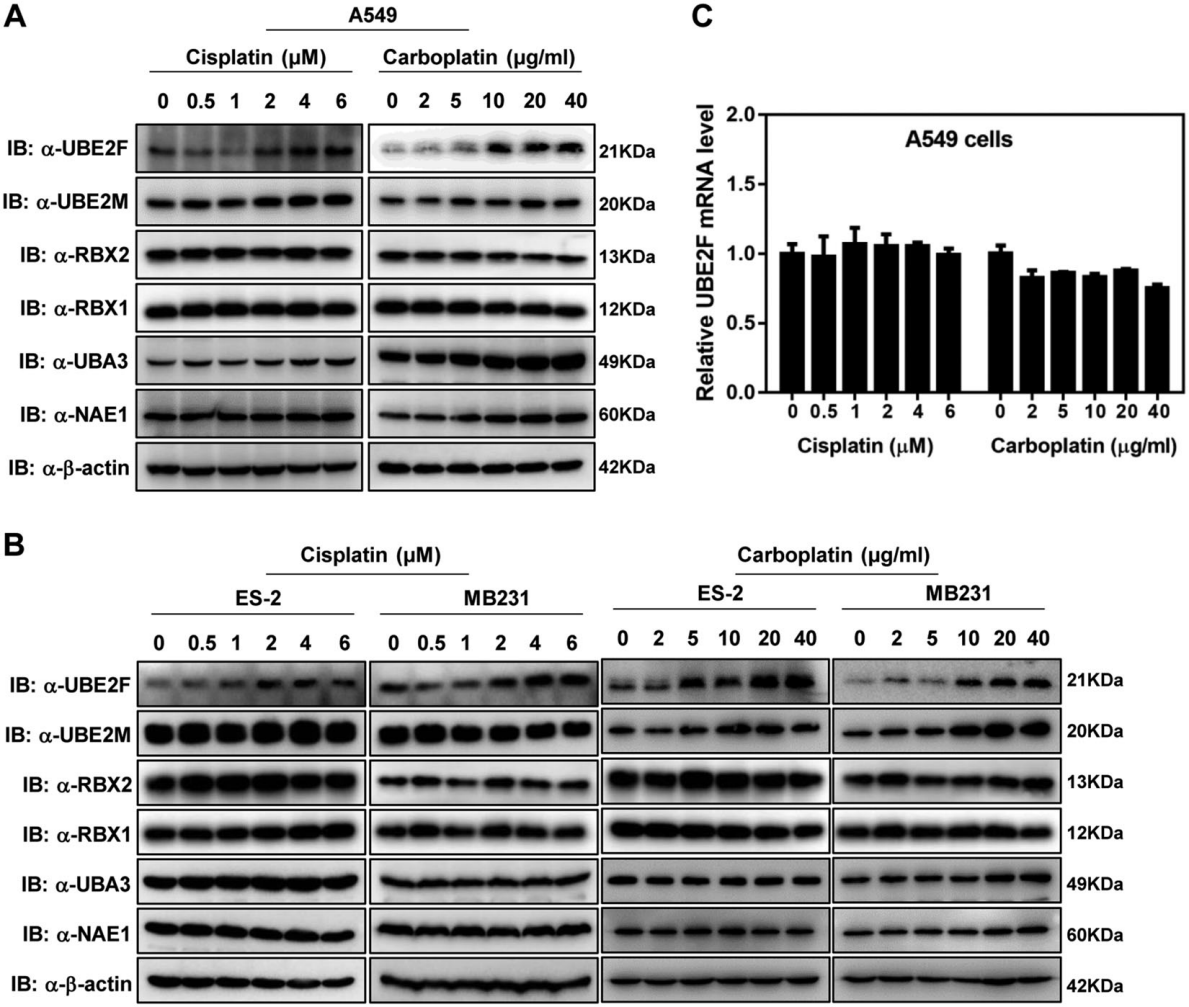
Platinum treatment promotes the accumulation of UBE2F. **A**, **B** Platinum treatment increases UBE2F protein levels. A549, ES-2, and MB231cells were treated with cisplatin or carboplatin at increasing concentrations versus PBS for 48 h as indicated. Protein levels of UBE2F were determined by western blot and normalized against β-actin. **C** Platinum treatment has no effect on the UBE2F mRNA levels. A549 cells were treated with cisplatin or carboplatin at increasing concentrations versus PBS for 48 h as indicated. mRNA levels of UBE2F were determined by RT-PCR and normalized against β-actin.

Platinum is a widely used chemotherapy drug in the treatment of many solid tumors by inducing apoptosis of tumor cells^24,25^. Giving the important role of the neddylation pathway in regulating apoptosis of cancer cells^7^, we first tested the effects of cisplatin or carboplatin treatment on the expression of neddylation components. Both cisplatin and carboplatin treatments in A549 and H1299, two human non-small cell lung cancer cell lines, led to the obvious accumulation of UBE2F in a dose-dependent manner, but not other neddylation components (e.g., E1: NAE1 and UBA3; E2: UBE2M; E3: RBX1 and RBX2) (Fig. 1A and Supplementary Fig. 1A). Similar results were observed in ES-2 (an ovarian cancer cell line) and MB231 (a breast cancer cell line) (Fig. 1B). Interestingly, treatment with cisplatin or carboplatin did not affect the mRNA levels of UBE2F (Fig. 1C and Supplementary Fig. 1B), indicating that platinum-induced accumulation of UBE2F is not related to the transcriptional regulation, but to the protein stability.

**Fig. 2:**
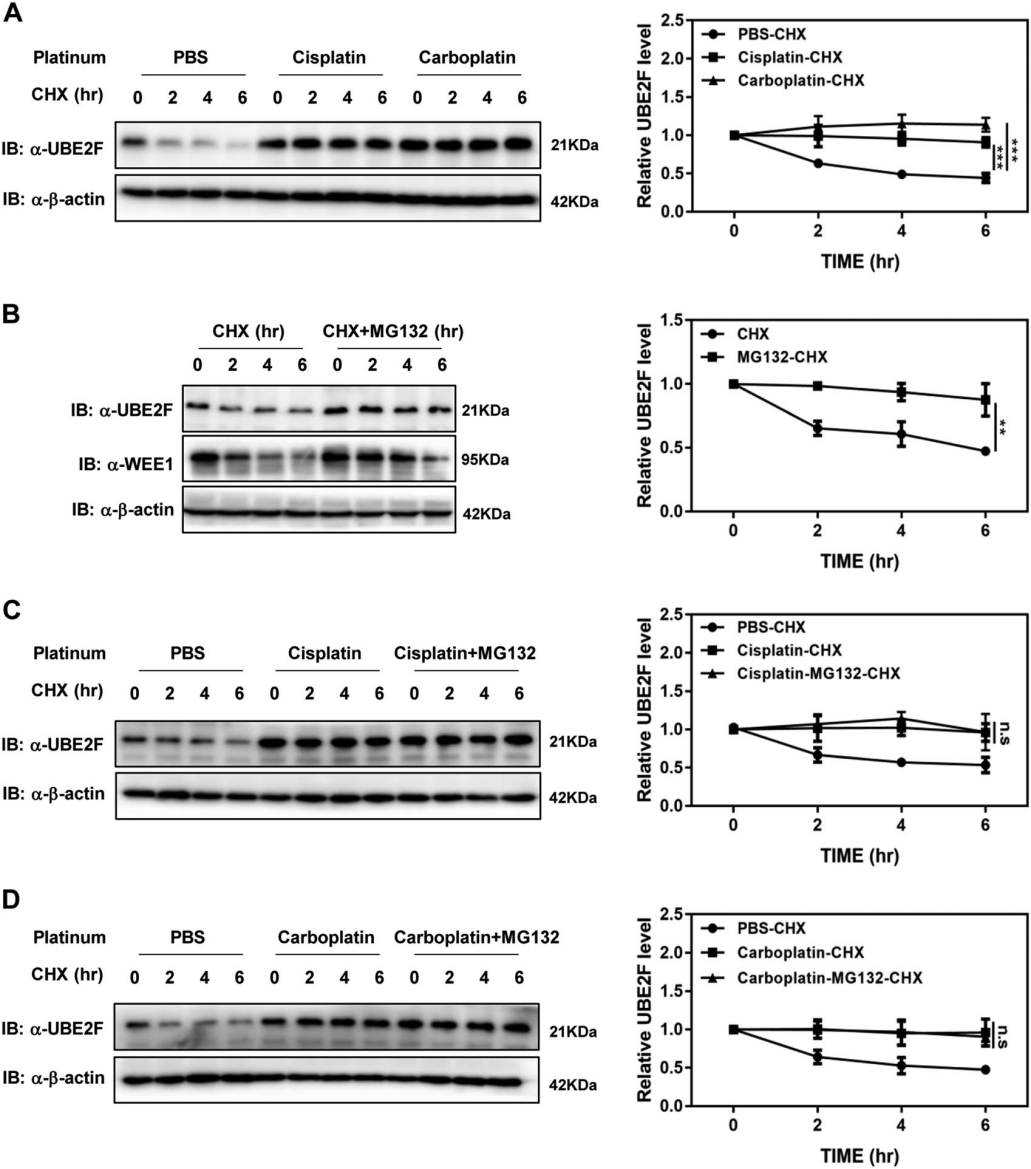
Platinum treatment causes the accumulation of UBE2F via the inhibition of proteasome-mediated protein degradation. **A** Platinum treatment extends the half-life of UBE2F. A549 cells were treated with cisplatin (4 μΜ) or carboplatin (20 μg/mL) versus PBS for 48 h, followed with 50 μg/mL CHX at the indicated time. **B** MG-132 extends the half-life of UBE2F. A549 cells were treated with 10 μM MG-132 versus DMSO in combination with 50 μg/mL CHX for the indicated time. **C**, **D** MG-132 could not further extend the half-life of UBE2F in the presence of platinum. A549 cells were treated with PBS, cisplatin (4 μΜ) or carboplatin (20 μg/mL) for 48 h, followed with 10 μM MG-132 versus DMSO in combination with 50 μg/mL CHX for the indicated time. The cell lysate was subjected to immunoblotting using antibodies against UBE2F with β-actin as a loading control. The half-life of UBE2F was calculated by mean value from three independent experiments. Shown are average values with standard deviation (s.d.). ^**^P < 0.001 and ^***^P < 0.0001 for the indicated comparison. n.s. not significant.

We next set out to examine the effect of protein synthesis inhibitor cycloheximide (CHX) on platinum-induced UBE2F accumulation. In A549 cells treated with CHX, we found that UBE2F is an unstable protein with a half-life of ~4 h. Interestingly, platinum obviously blocked CHX-triggered UBE2F protein decrease (Fig. 2A), supporting that platinum treatment suppresses the degradation of UBE2F. Proteasome and lysosome pathways are two major protein clearance systems in eukaryotic cells. When A549 cells were treated with the proteasome inhibitor MG-132, we observed an increase in the steady-state levels of UBE2F protein (Fig. 2B). However, lysosome inhibitors, such as chloroquine and bafilomycin A1, had no effect on the half-life of UBE2F protein (Supplementary Fig. 2). Moreover, MG-132 treatment could not further increase the steady-state levels of UBE2F protein in the presence of cisplatin or carboplatin (Fig. 2C, D), supporting that platinum treatment causes the accumulation of UBE2F via the inhibition of proteasome-mediated protein degradation.

### RBX1 negatively regulates UBE2F protein levels

**Fig. 3:**
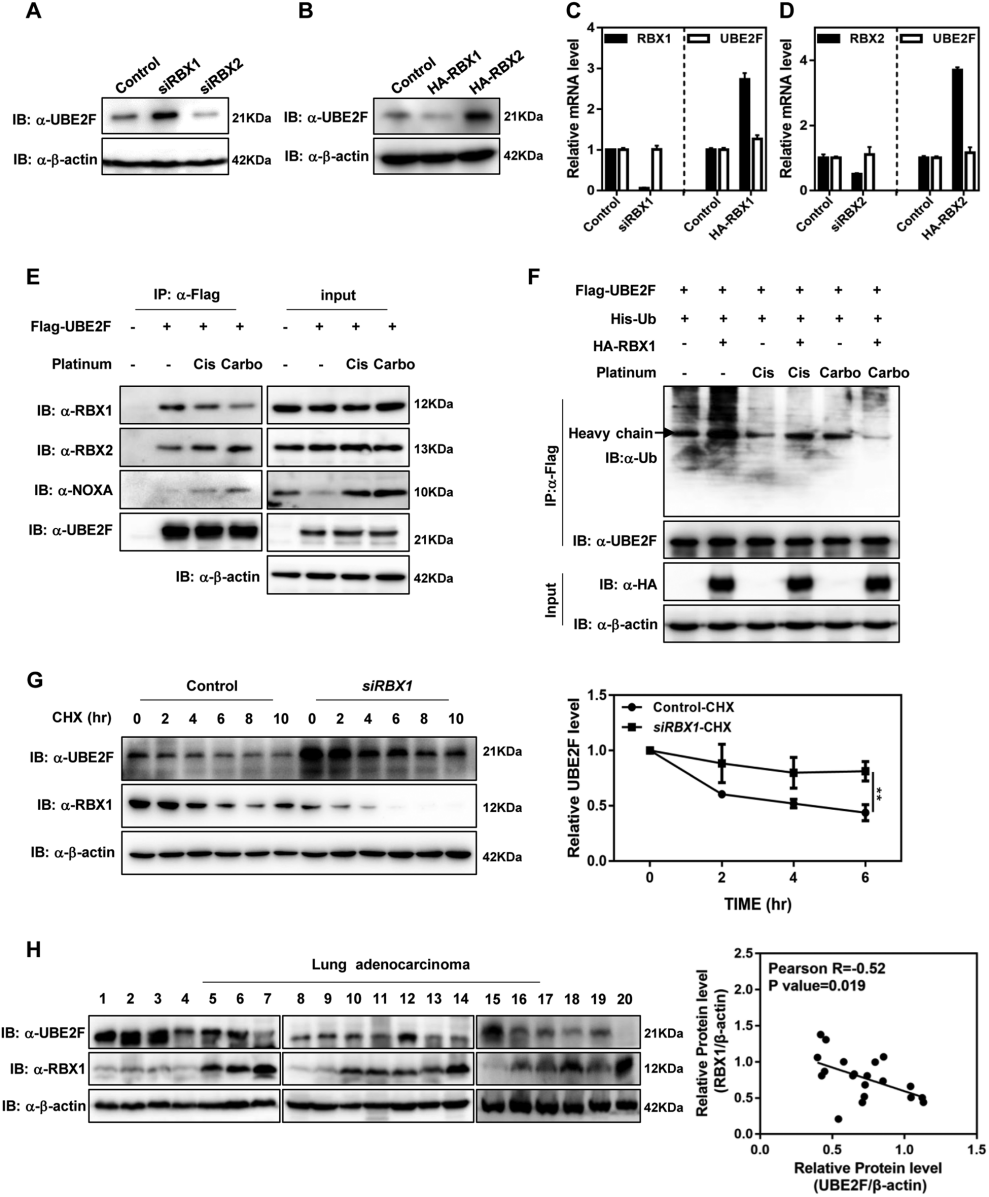
RBX1 negatively regulates UBE2F protein levels. **A**, **B** UBE2F protein levels are decreased by RBX1 and increased by RBX2. H1299 cells were transfected with siRNAs targeting RBX1/2 or indicated plasmids. Protein levels of UBE2F were determined by western blot and normalized against β-actin. **C**, **D** RBX1 and RBX2 have little or no effect on the mRNA levels of UBE2F. H1299 cells were transfected with siRNAs targeting RBX1/2 or indicated plasmids. mRNA levels of UBE2F, RBX1, and RBX2 were determined by RT-PCR and normalized against β-actin. **E** Platinum treatment impairs the protein interaction between UBE2F and RBX1. H1299 cells were transfected with indicated plasmids and then treated with cisplatin (Cis) or carboplatin (Carbo) for 48 h. UBE2F proteins were purified by Flag beads, and their protein interactions with endogenous RBX1, RBX2, and NOXA were determined by western blot. **F** Platinum treatment inhibits RBX1-mediated UBE2F poly-ubiquitylation. H1299 cells were co-transfected with indicated plasmids and then treated with cisplatin (Cis) or carboplatin (Carbo) for 48 h. UBE2F proteins were purified by Flag beads, and poly-ubiquitylation of UBE2F was determined by western blot. **G** Downregulation of RBX1 extends the half-life of UBE2F. A549 cells were transfected with control or RBX1 siRNA for 72 h and then treated with 50 μg/mL CHX at the indicated time. The half-life of UBE2F was calculated by mean value from three independent experiments. Shown are average values with standard deviation (s.d.). ^**^P < 0.001 for the indicated comparison. **H** UBE2F exerts a negative correlation with RBX1 in lung adenocarcinoma. 20 lung adenocarcinoma tissues were lysed. The UBE2F and RBX1 protein levels were compared against β-actin by western blot.

As noted in the introduction, CRLs are responsible for ubiquitylation and degradation of about 20% of proteasome-regulated proteins^12,13^. To determine whether CRL is involved in the degradation of UBE2F, we silenced all the cullins individually in A549 cells using small interfering RNA (*siRNA*), and found that in all six cullins, CUL-1, CUL3, and CUL4A knockdown increased UBE2F protein levels in varying degrees, while CUL4B and CUL-5 knockdown decreased UBE2F protein levels (Supplementary Fig. 3A). It has been previously reported that CUL3 promotes UBE2F degradation^23^, but our data shows that CUL-1 also has an important role in the accumulation of UBE2F (Supplementary Fig. 3A, B). Since RBX1 and RBX2 serve as essential components of CRL1–4 and CRL5, respectively^26,27^, we then determined the effect of both RBX1 and RBX2 on UBE2F levels. Our data demonstrated that UBE2F protein levels were increased by RBX1 knockdown, and were decreased by RBX1 overexpression; RBX2 had the opposite role (Fig. 3A, B). The knockdown or overexpression efficiency of RBX1 and RBX2 was determined by real-time PCR (Fig. 3C, D). Moreover, both RBX1 and RBX2 had little or no effect on the mRNA levels of UBE2F (Fig. 3C, D), indicating that the effect of RBX1 or RBX2 on UBE2F occurs at the post-translational level. Next, we determined the effect of platinum on the binding of UBE2F–RBX1 and UBE2F–RBX2 and found that cisplatin (Cis) or carboplatin (Carbo) treatment impaired the binding of UBE2F–RBX1, but promoted the binding of UBE2F–RBX2 (Fig. 3E). Given that UBE2F and RBX2 are neddylation E2 and E3 for CUL-5 neddylation, respectively, it is likely that they form complexes that stabilize UBE2F upon platinum treatment^22^. The weakened UBE2F–RBX1 interaction suggests that platinum treatment may promote UBE2F accumulation partially via blocking the RBX1–UBE2F degradation complex. Moreover, NOXA has been previously reported to bind with UBE2F and be subjected to poly-ubiquitylation by CRL5 for proteasomal degradation^22^. We found that cisplatin or carboplatin treatment increased UBE2F–NOXA interaction (Fig. 3E), which might trigger the degradation of NOXA to protect cells from apoptosis induced by platinum.

We then focused our study on RBX1 and determined its role in modulating platinum-induced UBE2F accumulation. Transiently co-overexpressing Flag-UBE2F, His-Ub, and HA-RBX1, we found that RBX1 overexpression promoted the poly-ubiquitylation of UBE2F, whereas cisplatin (Cis) or carboplatin (Carbo) treatment impaired it, indicating an RBX1-dependent manner (Fig. 3F). Supporting this notion, RBX1 knockdown significantly delayed the UBE2F turnover and extended the protein half-life of UBE2F (Fig. 3G). To examine the correlation of UBE2F and RBX1 expressions in clinical samples of human lung adenocarcinoma, we collected a total of 20 lung adenocarcinoma tissues and performed a direct immunoblotting analysis to determine the protein levels of UBE2F and RBX1. Using the Pearson correlation methods, we found that the protein levels of UBE2F vs. RBX1 exerted a significantly negative correlation in 20 lung adenocarcinoma samples (Pearson *R* = −0.52, *P* = 0.019) (Fig. 3H). Taken together, these findings indicate that platinum treatment induces the accumulation of UBE2F, at least partially through suppressing RBX1-mediated proteasome degradation of UBE2F.

### Inhibition of UBE2F sensitizes lung cancer cells to platinum

**Fig. 4:**
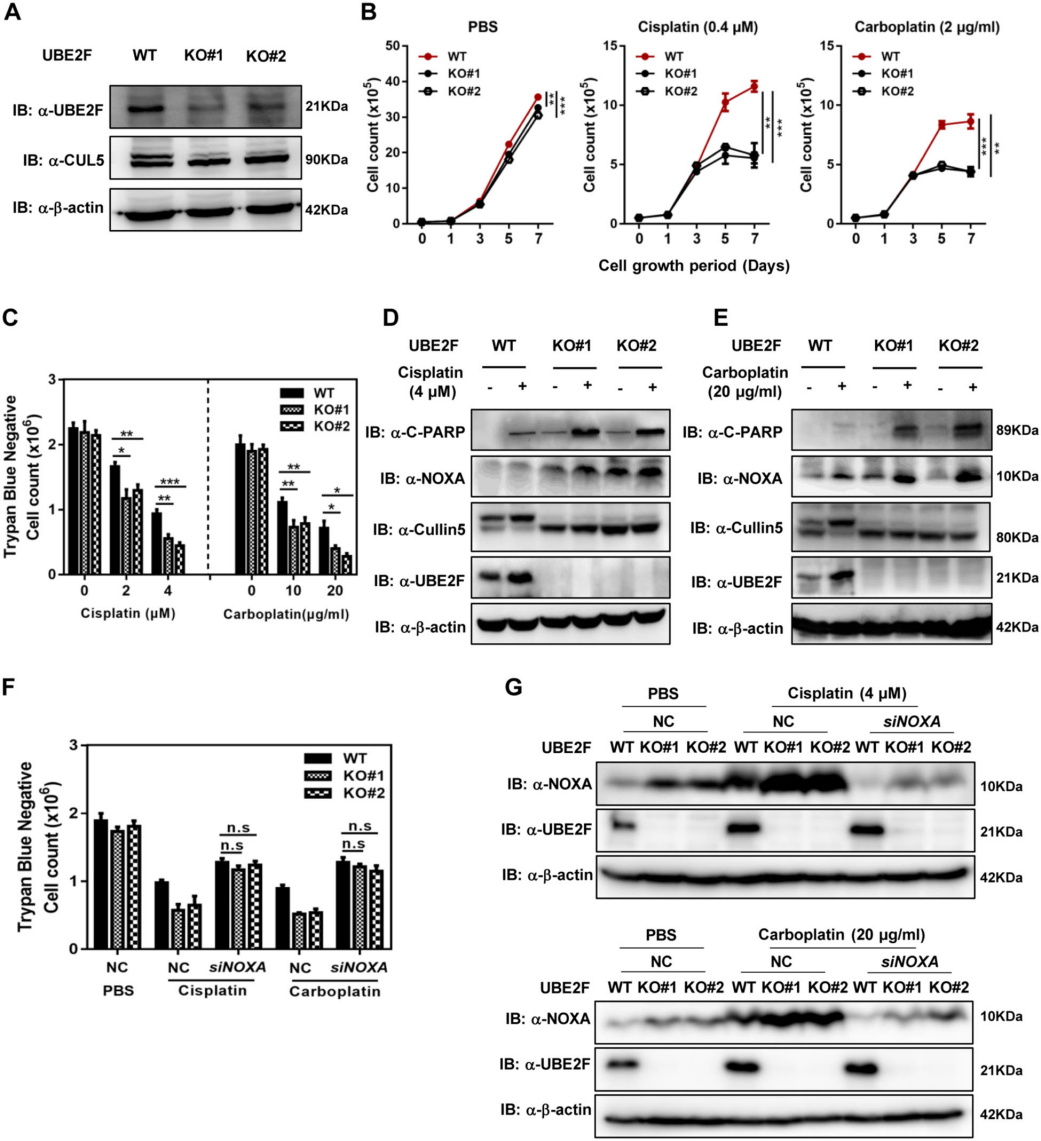
Inhibition of UBE2F sensitizes lung cancer cells to platinum. **A** Establishment of UBE2F KO A549 stable cells. A549 cells with stable UBE2F knockout were generated by retrovirus infection, and the UBE2F protein levels were determined by western blot. **B** UBE2F protects lung cancer cells from platinum-induced growth inhibition. A549 WT and UBE2F KO cells were seeded in 6-well plates at a density of 50,000 cells/well, followed by treatment of low concentration of platinum as indicated for 7 days, and the remaining living cells were trypsinized and counted by Nexcelom. **C** UBE2F protects lung cancer cells from platinum-induced cell death. A549 WT and UBE2F KO cells were seeded in 6-well plates at a density of 500,000 cells/well, followed by treatment of high concentration of platinum as indicated for 48 h and the remaining living cells were trypsinized and counted by Nexcelom. **D**, **E** UBE2F deficiency leads to lung cancer cells more sensitive to platinum. A549 WT and UBE2F KO cells were seeded in 6-well plates at a density of 500,000 cells/well, followed by treatment of platinum as indicated for 48 h. The expression levels of PARP and NOXA were determined by western blot. **F**, **G** NOXA has an essential role in UBE2F-mediated apoptotic resistance to platinum. A549 WT and UBE2F KO cells were transfected with siRNA targeting *NOXA*. Cells were seeded in 6-well plates at a density of 500,000 cells/well, followed by treatment of high concentration of platinum as indicated for 48 h and the remaining living cells were trypsinized and counted by Nexcelom. The expression levels of NOXA were determined by western blot. Shown are average values with standard deviation (s.d.). ^*^P < 0.05, ^**^P < 0.001, and ^***^P < 0.001 for the indicated comparison. n.s. not significant.

Previous studies have shown that UBE2F acts as an anti-apoptotic protein via negatively regulating the protein levels of NOXA^22,23^, leading us to investigate whether the upregulation of UBE2F is an important way for cancer cells to escape platinum-induced cell apoptosis. To this end, we generated stable A549 cells with UBE2F knockout by CRISPR/Cas9 system to determine whether UBE2F deletion allows for the platinum sensitivity for lung cancer cells. Two independent UBE2F knockout cell pools were obtained with two independent guide sequences and were verified by immunoblotting, in line with lower levels of CUL-5 neddylation (Fig. 4A). UBE2F knockout (KO) mildly affected the proliferation of lung cancer cells, but sharply promoted the inhibition of proliferation induced by cisplatin or carboplatin at a low dose, compared with wild-type (WT) since day 5 of giving drugs (Fig. 4B). Moreover, UBE2F knockout also sensitized cells to cell death induced by a high dose of cisplatin or carboplatin, positively correlated with the platinum dose (Fig. 4C). In accord, UBE2F knockout cells remarkedly exhibited higher levels of cleaved PARP and NOXA, two indicators of apoptosis, when subjected to cisplatin or carboplatin (Fig. 4D, E). Next, we utilized siRNA to deplete the *NOXA* gene, and found that the effect of UBE2F deficiency on the cell death upon cisplatin or carboplatin treatment was diminished by the depletion of NOXA (Fig. 4F, G). Given that pro-apoptotic protein, NOXA is a well-known substrate of UBE2F-CRL5, these results indicate that UBE2F deletion delays the degradation of NOXA, and thus contributes to lung cancer cells sensitive to platinum.

### UBE2F protects lung cancer cells from platinum chemotherapy in vivo

**Fig. 5:**
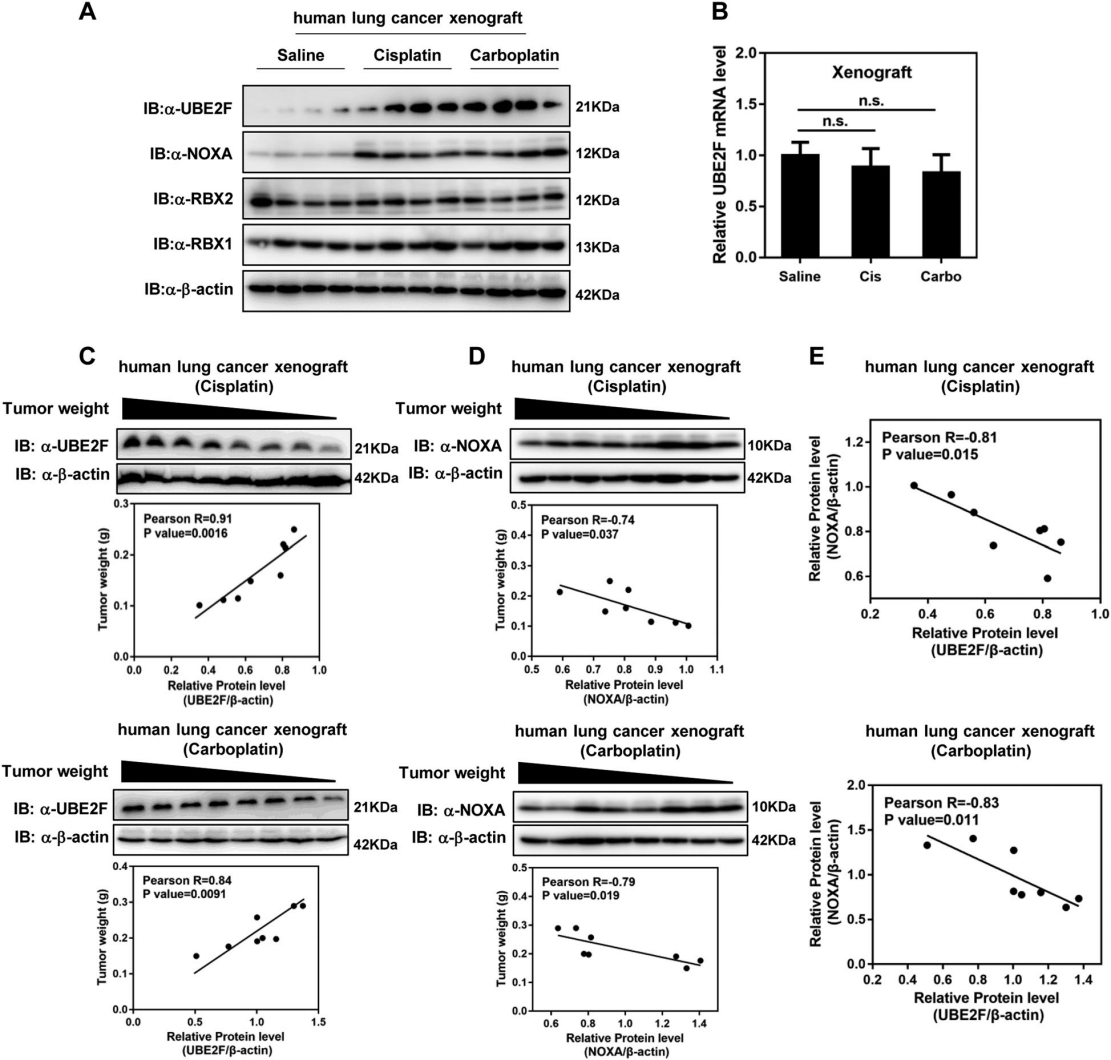
UBE2F in platinum-treated xenografts is significantly and inversely correlated with tumor weight and NOXA, respectively. **A** Platinum treatment increases protein levels of UBE2F and NOXA in xenograft tumors. A549 cells (2 × 10^6^) were injected subcutaneously into the flanks of nude mice. When the size of the tumor reaches about 20 mm^2^, mice were intraperitoneally injected with saline, cisplatin (4 mg/kg, once every 4 days, three cycles), or carboplatin (25 mg/kg, once every 2 days, six cycles). Protein levels of UBE2F, NOXA, RBX1, and RBX2 were determined by western blot and normalized against β-actin. **B** Platinum treatment has no effect on the UBE2F mRNA levels in xenograft tumors. Nude mice with xenografts were treated as in **A**. mRNA levels of UBE2F were determined by RT-PCR and normalized against β-actin. **C**, **D** Protein levels of UBE2F and NOXA are positively and negatively correlated with the weight of the xenograft tumors subjected to platinum, respectively. Nude mice with xenografts were treated as in **A**. At the end of the experiments, tumor xenografts were measured for weight. **E** UBE2F is inversely correlated with NOXA in xenograft tumors subjected to platinum.

Next, we performed a xenograft model to further elucidate the effect of platinum treatment on the expression of UBE2F in vivo. As shown, the protein levels of UBE2F and NOXA, but not RBX1 and RBX2, were obviously increased in tumors from nude mice injected with cisplatin or carboplatin than that from mice with saline injection (Fig. 5A). Consistent with in vitro experiments, the mRNA levels of UBE2F were not affected upon cisplatin or carboplatin treatment (Fig. 5B). These findings support that the accumulation of UBE2F induced by platinum is mainly due to the increased protein stability in vivo.

Further analysis showed that, in tumors from mice injected with cisplatin or carboplatin, the protein levels of UBE2F and NOXA were positively and negatively correlated with tumor weight, respectively (UBE2F: cisplatin injection, Pearson *R* = 0.91, *P* = 0.0016; carboplatin injection, Pearson *R* = 0.84, *P* = 0.0091. NOXA: cisplatin injection, Pearson *R* = −0.74, *P* = 0.037; carboplatin injection, Pearson *R* = −0.79, *P* = 0.019) (Fig. 5C, D). Moreover, using the Pearson correlation methods, we found that the expression of UBE2F/β-actin vs. NOXA/β-actin exerted significantly negative correlation (cisplatin injection, Pearson *R* = −0.81, *P* = 0.015; carboplatin injection, Pearson *R* = −0.83, *P* = 0.011) (Fig. 5E). Given that transcriptional activation of NOXA is critical for cisplatin-induced apoptosis^28,29^, the accumulation of UBE2F might have a potential role in the resistance of platinum drugs via inhibiting NOXA in lung cancer chemotherapy.

**Fig. 6:**
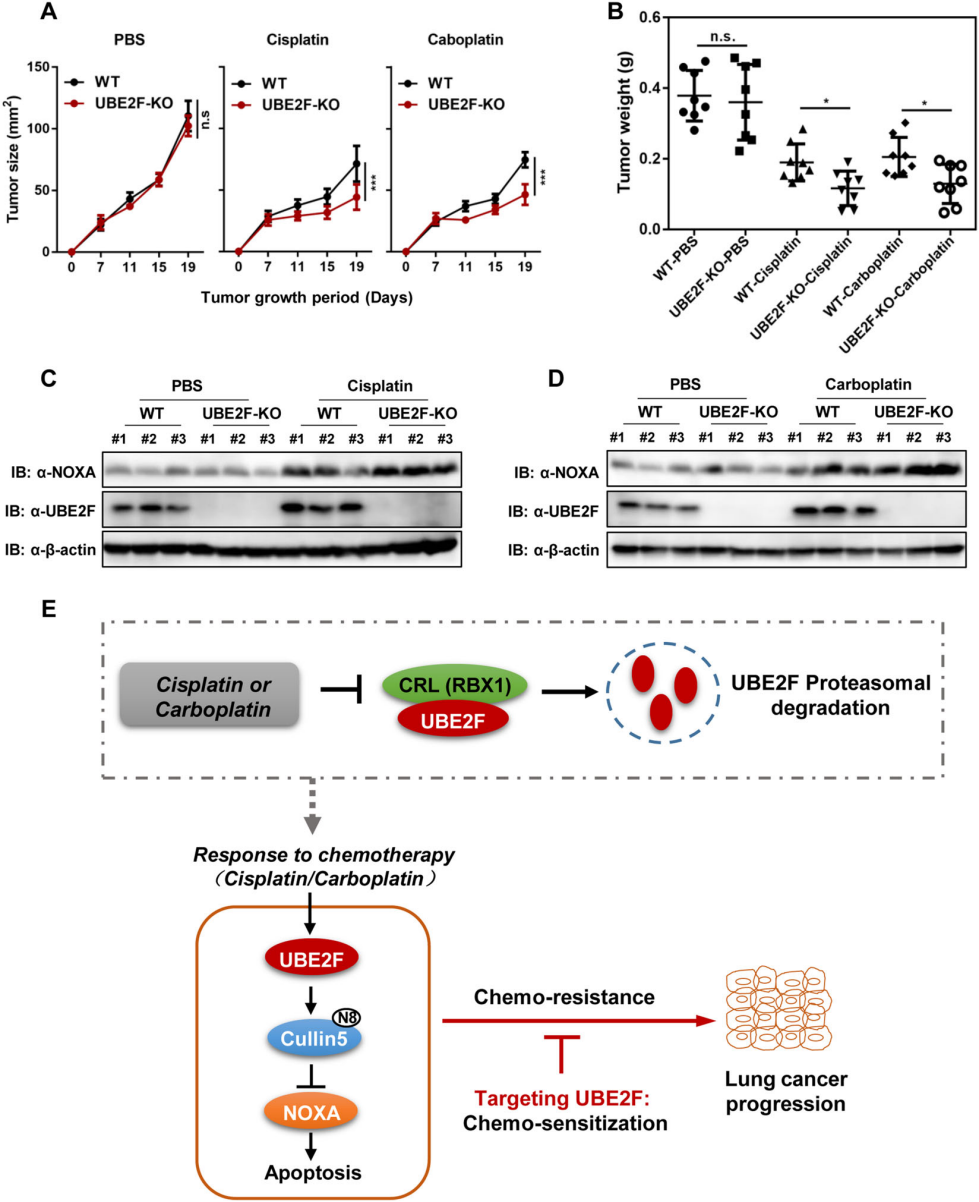
UBE2F protects lung cancer cells from platinum chemotherapy in vivo. **A**–**D** UBE2F protects tumors from platinum-induced growth inhibition. Nude mice with xenografts were treated as in Fig. 5A. Tumor xenografts were measured for size (**A**) and weight (**B**). The expression levels of UBE2F and NOXA were determined by western blot (**C**, **D**). Shown are average values with standard deviation (s.d.). ^*^P < 0.05 and ^***^P < 0.001 for the indicated comparison; n.s. not significant. **E** A working model illustrating the role of UBE2F in platinum sensitivity. Shown is a working model depicting that upregulation of neddylation E2 UBE2F is an important way for cancer cells to escape platinum-induced cell apoptosis, which is supported by the deletion of UBE2F promotes platinum-induced apoptosis. Platinum impaired the interaction of the UBE2F–RBX1 complex and subsequently inhibited its degradation, resulting in the degradation of NOXA and consequently inducing apoptotic resistance to platinum chemotherapy.

We then performed xenograft experiments using UBE2F WT and KO lung cancer cells and compared the in vivo response of these two cell lines after cisplatin or carboplatin injection. As shown, no significant difference in the tumor sizes was found in the xenografts of UBE2F WT and KO lung cancer cells under non-treatment conditions (upon saline injection) (Fig. 6A). However, the tumor sizes in nude mice injected with cells UBE2F KO were significantly smaller than these from mice injected WT cells under chemotherapy condition (upon cisplatin or carboplatin injection) (tumor size at 21 days: cisplatin, 44.2 vs. 71.3 mm^2^ for UBE2F KO vs. WT, *P* < 0.0001; carboplatin, 46.4 vs. 74.7 mm^2^ for UBE2F KO vs. WT, *P* < 0.0001) (Fig. 6A). The tumor weight in nude mice injected UBE2F KO cells was also significantly (*P* < 0.05) lighter than that from mice injected WT cells after cisplatin or carboplatin injection (Fig. 6B). In accord with tumor growth inhibition, UBE2F knockout cells remarkedly exhibited higher levels of NOXA when subjected to cisplatin or carboplatin (Fig. 6C, D). Taken together, these data support a crucial role of UBE2F in platinum-based chemosensitivity and suggest a possible benefit of combining UBE2F inhibitors with platinum therapy.

## Discussion

Platinum is a widely used first-line chemotherapy in treating multiple human malignancies by inducing cancer cells apoptosis^24^. Unfortunately, primary or secondary platinum resistance leads to relapse and therapeutic failure, enabling the development of platinum-sensitization strategies to be of great clinical significance^24^. In the present study, we found that the upregulation of neddylation E2 UBE2F is an important way for cancer cells to escape platinum-induced cell apoptosis, which is supported by that the deletion of UBE2F promotes platinum-induced apoptosis (Fig. 6E). Platinum-induced accumulation of UBE2F expression results in the degradation of NOXA, consequently inducing apoptotic resistance to platinum chemotherapy. Importantly, besides lung cancer cells, multiple cancer cells, including ovarian cancer cells and breast cancer cells, all could upregulate the expression of UBE2F responding to platinum treatments, suggesting UBE2F may be a potentially universal drug target of platinum-sensitization. Of note, the expressions of other neddylation components (e.g., UBE2M) are not affected by platinum treatments. A very recent study reported that UBE2M is recruited to DNA-damage sites, and the deletion of UBE2M significantly sensitizes cells to IR or to other DNA-damaging agents^20,21^. These data demonstrate the complexity of neddylation pathway components regulating chemosensitivity, supporting the notion that the neddylation pathway serves as a promising anticancer target for a variety of chemo-resistant cancers. To the best of our knowledge, this is the first study to show regulation of the neddylation pathway functions on chemosensitivity at a protein level.

We uncover a previously unknown mechanism by which platinum treatment stimulates UBE2F accumulation: platinum impaired the interaction of UBE2F–RBX1 complex and subsequently inhibited its degradation. There is a question that how small molecule cisplatin or carboplatin disrupts the RBX1–UBE2F binding. A recent study showed that gossypol, a natural compound derived from cotton seed, blocks the neddylation of both CUL-5 and CUL-1 through direct binding to RBX1–CUL-1 or RBX2–CUL-5 complex, indicating that a small molecular likely disrupts protein-protein interaction^30^. In this study, platinum or its intermediates might be located at the protein-protein interface between UBE2F and RBX1 to disrupt the UBE2F–RBX1 binding, which in turn may facilitate the binding of UBE2F–RBX2. To illuminate the hypothesis, computer modeling analysis, and more biochemical experiments will be needed.

Since RBX1 serves as an essential component of both CRL1 and CRL3, the deletion of RBX1 is more conducive to UBE2F accumulation. We observed a reduction in RBX1 expression is in parallel with an increase in UBE2F expression in lung adenocarcinoma tissues. However, it is difficult to determine whether platinum chemotherapy reversely regulates the expression of UBE2F and RBX1, because most patients with NSCLC rarely undergo surgery after chemotherapy. Substrate specificity of CRL1 E3 ligase is determined largely by the F-box protein, which usually recognizes phosphorylated target protein^31,32^. Thus, further exploration will be needed to illuminate which F-box protein negatively regulates the stability of UBE2F in response to platinum chemotherapy and whether UBE2F is a phosphorylated protein.

In summary, our study uncovers a previously unknown function of UBE2F in platinum chemosensitivity and a new mechanism that regulates UBE2F stability in response to platinum chemotherapy. We anticipate that these findings will trigger future investigation on the role of UBE2F in the chemosensitivity of other drugs in different tumors and the development of UBE2F-targeted drugs.

## Supplementary information

Supplementary Figure Legends

Supplementary Figure 1

Supplementary Figure 2

Supplementary Figure 3
