# Supplementary material for: Induction of NEDD8-conjugating enzyme E2 UBE2F by platinum protects lung cancer cells from apoptosis and confers to platinum-insensitivity
Source: Cell Death Dis. 2020 Nov 12;11(11):975. doi: 10.1038/s41419-020-03184-4 (PMC7665193; doi:10.1038/s41419-020-03184-4)
Supplement: Supplementary file 1 — Supplementary Figure Legends [file 41419_2020_3184_MOESM1_ESM.docx]

**Supplementary Figure Legends**

**Supplementary Fig. 1 Platinum treatment promotes the accumulation of UBE2F in H1299 cells**

(A) Platinum treatments increase endogenous UBE2F protein levels in H1299 cells. H1299 cells were treated with cisplatin or carboplatin at increasing concentrations versus PBS for 48 hours as indicated. Protein levels of UBE2F were determined by western blot and normalized against β-actin.

(B) Platinum treatment has no effect on the UBE2F mRNA levels in H1299 cells. H1299 cells were treated with cisplatin or carboplatin at increasing concentrations versus PBS for 48 hours as indicated. mRNA levels of UBE2F were determined by RT-PCR and normalized against β-actin.

**Supplementary Fig. 2 Lysosome inhibitors have no effect on the half-life of UBE2F protein**

A549 cells were treated with chloroquine (10 μΜ) or bafilomycin A1 (20 nM) versus PBS for 72 h, and then treated with 50 μg/mL CHX at indicated time before subjected to immunoblotting using antibodies against UBE2F with β-actin as a loading control.

**Supplementary Fig. 3 CUL1 negatively regulates UBE2F protein levels**

(A) Down-regulation of Cullin 1 induces the accumulation of UBE2F. A549 cells were transfected with control (Ctrl) or Cullins siRNA for 72 h and harvested for immunoblotting using antibodies against UBE2F and different Cullins with β-actin as a loading control.

(B) Down-regulation of CUL1 extends the half-life of UBE2F. A549 cells were transfected with control (Ctrl) or CUL1 siRNA for 72 h and then treated with 50 μg/mL CHX at indicated time before subjected to immunoblotting using antibodies against UBE2F with β-actin as a loading control.
