# Supplementary figures and images for: Induction of NEDD8-conjugating enzyme E2 UBE2F by platinum protects lung cancer cells from apoptosis and confers to platinum-insensitivity
Source: Cell Death Dis. 2020 Nov 12;11(11):975. doi: 10.1038/s41419-020-03184-4 (PMC7665193; doi:10.1038/s41419-020-03184-4)

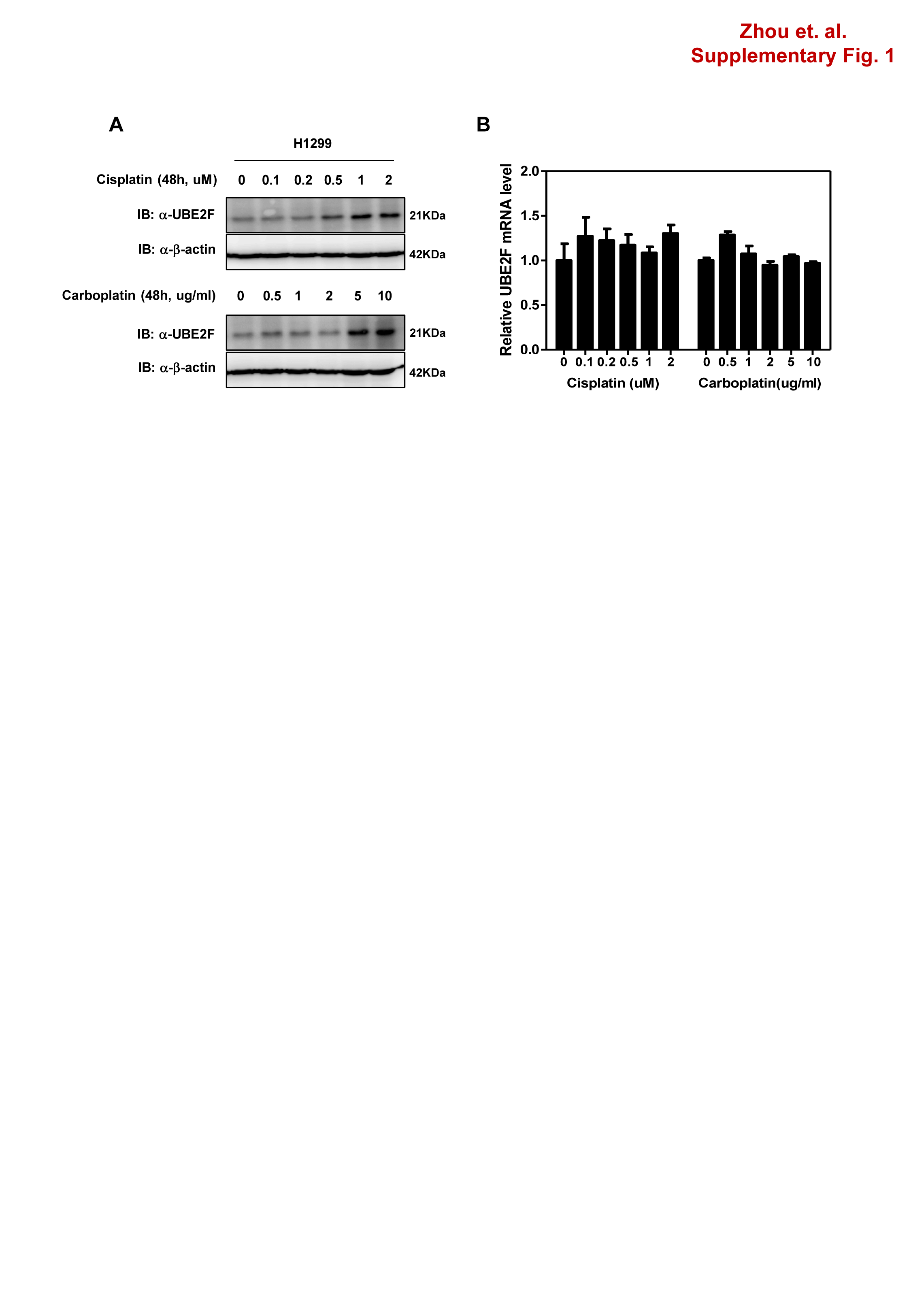

Supplement: Supplementary file 2 — Supplementary Figure 1 [file 41419_2020_3184_MOESM2_ESM.tif]

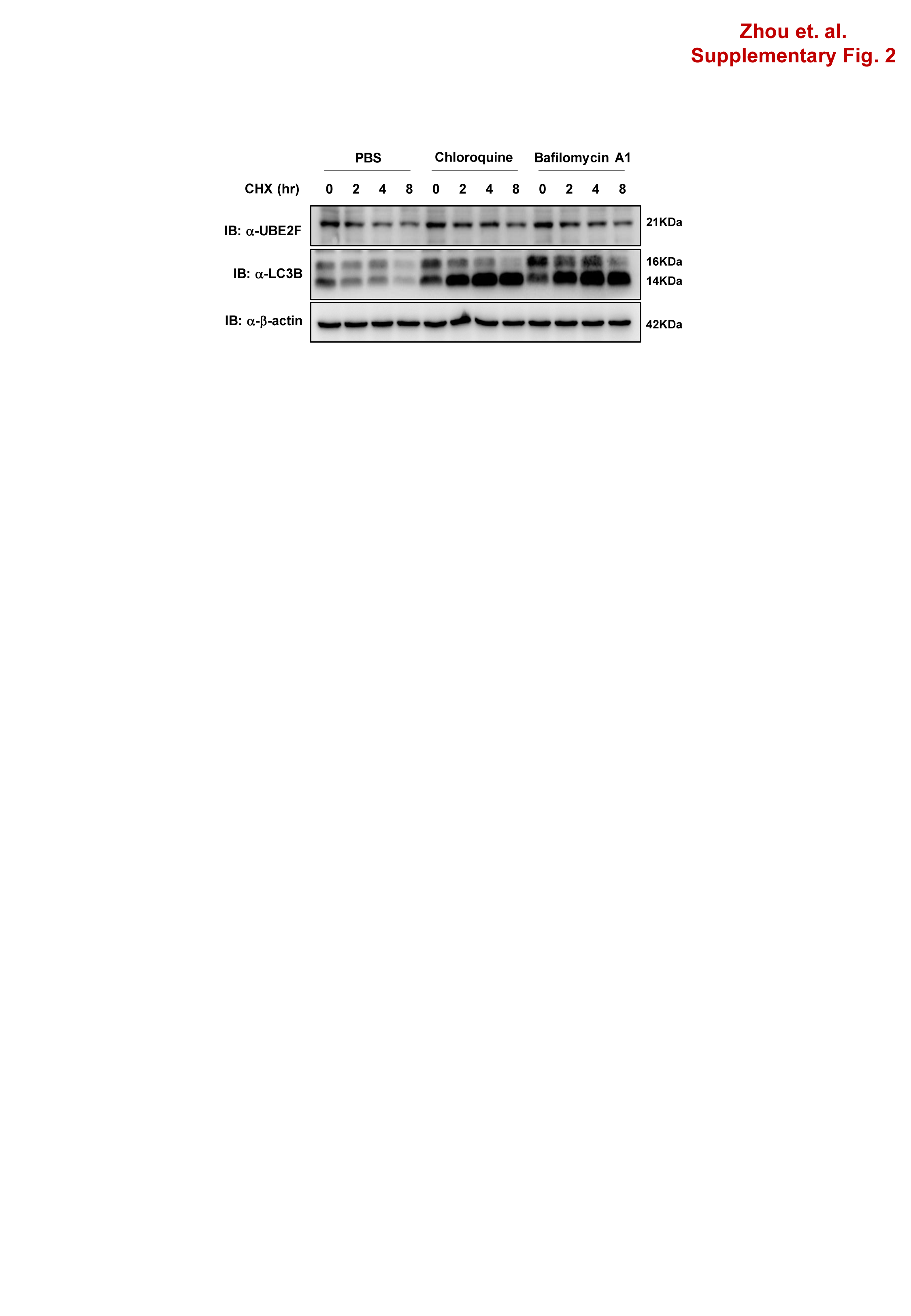

Supplement: Supplementary file 3 — Supplementary Figure 2 [file 41419_2020_3184_MOESM3_ESM.tif]

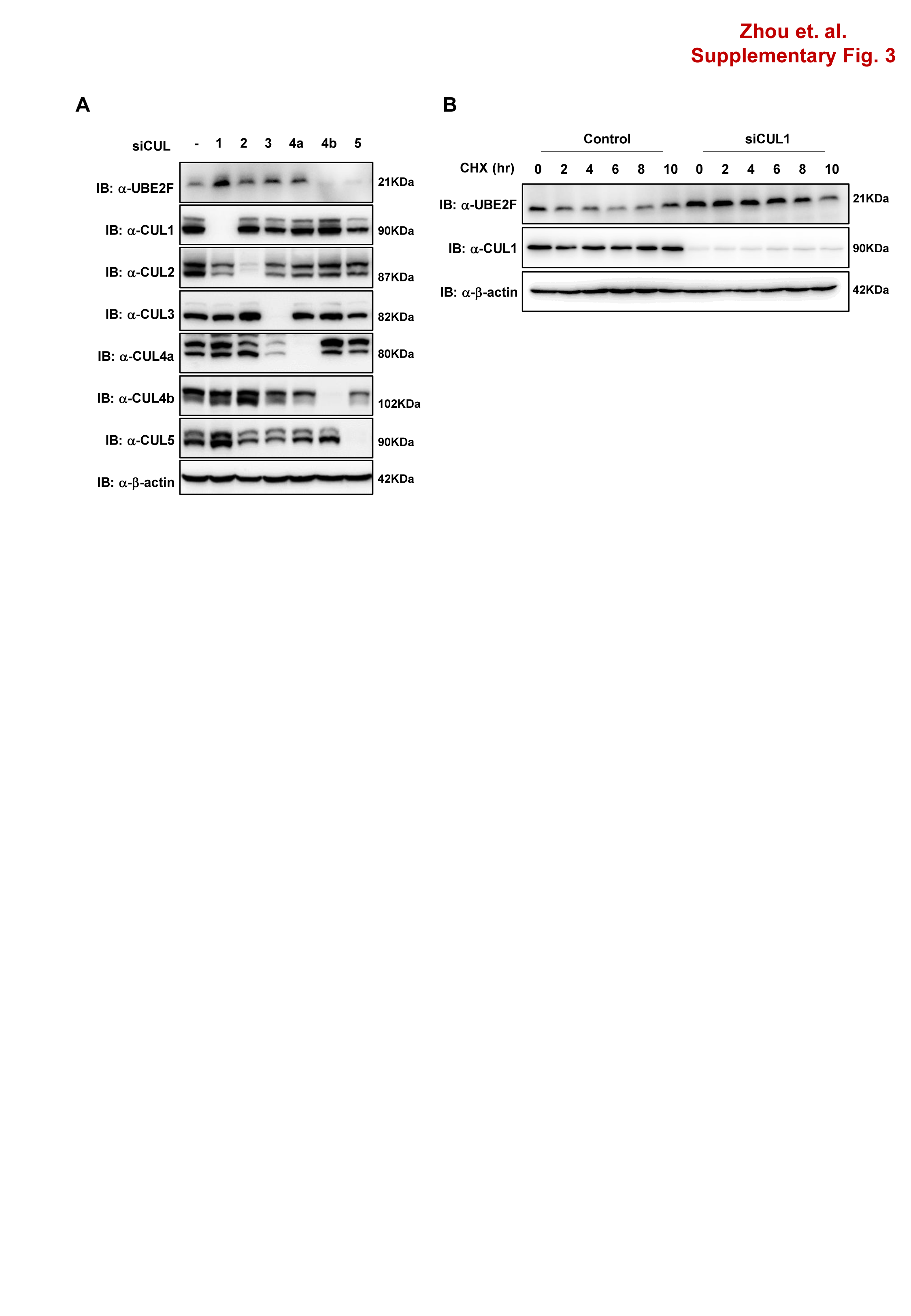

Supplement: Supplementary file 4 — Supplementary Figure 3 [file 41419_2020_3184_MOESM4_ESM.tif]
